# Anemia in Patients with Severe Aortic Stenosis

**DOI:** 10.1038/s41598-018-36066-z

**Published:** 2019-02-13

**Authors:** Kazuya Nagao, Tomohiko Taniguchi, Takeshi Morimoto, Hiroki Shiomi, Kenji Ando, Norio Kanamori, Koichiro Murata, Takeshi Kitai, Yuichi Kawase, Chisato Izumi, Makoto Miyake, Hirokazu Mitsuoka, Masashi Kato, Yutaka Hirano, Shintaro Matsuda, Tsukasa Inada, Tomoyuki Murakami, Yasuyo Takeuchi, Keiichiro Yamane, Mamoru Toyofuku, Mitsuru Ishii, Eri Minamino-Muta, Takao Kato, Moriaki Inoko, Tomoyuki Ikeda, Akihiro Komasa, Katsuhisa Ishii, Kozo Hotta, Nobuya Higashitani, Yoshihiro Kato, Yasutaka Inuzuka, Chiyo Maeda, Toshikazu Jinnai, Yuko Morikami, Naritatsu Saito, Kenji Minatoya, Takeshi Kimura, CURRENT AS Registry Investigators, Naoki Takahashi, Kohei Fukuchi, Masao Imai, Junichi Tazaki, Toshiaki Toyota, Hirooki Higami, Tetsuma Kawaji, Shinichi Shirai, Kengo Kourai, Takeshi Arita, Shiro Miura, Kyohei Yamaji, Tomoya Onodera, Yutaka Furukawa, Kitae Kim, Kazushige Kadota, Keiichiro Iwasaki, Hiroshi Miyawaki, Ayumi Misao, Akimune Kuwayama, Masanobu Ohya, Takenobu Shimada, Hidewo Amano, Yoshihisa Nakagawa, Masashi Amano, Yusuke Takahashi, Yusuke Yoshikawa, Shunsuke Nishimura, Maiko Kuroda, Manabu Shirotani, Shinji Miki, Tetsu Mizoguchi, Takafumi Yokomatsu, Akihiro Kushiyama, Hidenori Yaku, Toshimitsu Watanabe, Shunichi Miyazaki, Teruki Takeda, Tomoko Sakaguchi, Keiko Maeda, Masayuki Yamaji, Maenaka Motoyoshi, Yutaka Tadano, Hiroki Sakamoto, Makoto Motooka, Ryusuke Nishikawa, Hiroshi Eizawa, Mitsunori Kawato, Minako Kinoshita, Kenji Aida, Takashi Tamura, Kousuke Takahashi, Euihong Ko, Masaharu Akao, Nobutoyo Masunaga, Hisashi Ogawa, Moritake Iguchi, Takashi Unoki, Kensuke Takabayashi, Yasuhiro Hamatani, Yugo Yamashita, Yoshihiro Himura, Yukihito Sato, Shuhei Tsuji, Takashi Konishi, Kouji Sogabe, Michiya Tachiiri, Yukiko Matsumura, Chihiro Ota, Ichiro Kouchi, Shigeru Ikeguchi, Soji Nishio, Jyunya Seki, Eiji Shinoda, Miho Yamada, Akira Kawamoto, Shoji Kitaguchi, Ryuzo Sakata, Mitsuo Matsuda, Sachiko Sugioka, Yuji Hiraoka, Michiya Hanyu, Fumio Yamazaki, Tadaaki Koyama, Tatsuhiko Komiya, Kazuo Yamanaka, Noboru Nishiwaki, Hiroyuki Nakajima, Motoaki Ohnaka, Hiroaki Osada, Katsuaki Meshii, Toshihiko Saga, Masahiko Onoe, Shogo Nakayama, Genichi Sakaguchi, Atsushi Iwakura, Kotaro Shiraga, Koji Ueyama, Keiichi Fujiwara, Atsushi Fukumoto, Senri Miwa, Junichiro Nishizawa, Mitsuru Kitano

**Affiliations:** 1 Cardiovascular Center, Osaka Red Cross Hospital, Osaka, Japan; 2 Cardiovascular Medicine, Kyoto University Graduate School of Medicine, Kyoto, Japan; 3 Department of Clinical Epidemiology, Hyogo College of Medicine, Nishinomiya, Japan; 4 Department of Cardiology, Kokura Memorial Hospital, Kokura, Japan; 5 Division of Cardiology, Shimada Municipal Hospital, Shimada, Japan; 6 Department of Cardiology, Shizuoka City Shizuoka Hospital, Shizuoka, Japan; 7 Department of Cardiovascular Medicine, Kobe City Medical Center General Hospital, Kobe, Japan; 8 Department of Cardiovascular Medicine, Kurashiki Central Hospital, Kurashiki, Japan; 9 Department of Cardiology, Tenri Hospital, Tenri, Japan; 10 Division of Cardiology, Nara Hospital, Kinki University Faculty of Medicine, Ikoma, Japan; 11 Department of Cardiology, Mitsubishi Kyoto Hospital, Kyoto, Japan; 12 Department of Cardiology, Kinki University Hospital, Osakasayama, Japan; 13 Department of Cardiology, Koto Memorial Hospital, Higashiomi, Japan; 14 Department of Cardiology, Shizuoka General Hospital, Shizuoka, Japan; 15 Department of Cardiology, Nishikobe Medical Center, Kobe, Japan; 16 Department of Cardiology, Japanese Red Cross Wakayama Medical Center, Wakayama, Japan; 17 Department of Cardiology, National Hospital Organization Kyoto Medical Center, Kyoto, Japan; 18 Cardiovascular Center, The Tazuke Kofukai Medical Research Institute, Kitano Hospital, Osaka, Japan; 19 Department of Cardiology, Hikone Municipal Hospital, Hikone, Japan; 20 Department of Cardiology, Kansai Electric Power Hospital, Osaka, Japan; 21 Department of Cardiology, Hyogo Prefectural Amagasaki General Medical Center, Amagasaki, Japan; 22 Department of Cardiology, Japanese Red Cross Otsu Hospital, Otsu, Japan; 23 Department of Cardiology, Saiseikai Noe Hospital, Osaka, Japan; 24 Department of Cardiology, Shiga Medical Center for Adults, Moriyama, Japan; 25 Department of Cardiology, Hamamatsu Rosai Hospital, Hamamatsu, Japan; 26 Department of Cardiology, Hirakata Kohsai Hospital, Hirakata, Japan; 27 Department of Cardiovascular Surgery, Kyoto University Graduate School of Medicine, Kyoto, Japan; 28 Department of Cardiology, Kishiwada City Hospital, Kishiwada, Japan; 29 Department of Cardiology, Rakuwakai Otowa Hospital, Kyoto, Japan; 30 Department of Cardiovascular Surgery, Kokura Memorial Hospital, Kitakyushu, Japan; 31 Department of Cardiovascular Surgery, Shizuoka City Shizuoka Hospital, Shizuoka, Japan; 32 Department of Cardiovascular Surgery, Kobe City Medical Center General Hospital, Kobe, Japan; 33 Department of Cardiovascular Surgery, Kurashiki Central Hospital, Kurashiki, Japan; 34 Department of Cardiovascular Surgery, Tenri Hospital, Tenri, Japan; 35 Department of Cardiovascular Surgery, Nara Hospital, Kinki University Faculty of Medicine, Ikoma, Japan; 36 Department of Cardiovascular Surgery, Mitsubishi Kyoto Hospital, Kyoto, Japan; 37 Department of Cardiovascular Surgery, Kinki University Hospital, Osakasayama, Japan; 38 Department of Cardiovascular Surgery, Kishiwada City Hospital, Kishiwada, Japan; 39 Department of Cardiovascular Surgery, Osaka Red Cross Hospital, Osaka, Japan; 40 Department of Cardiovascular Surgery, Shizuoka General Hospital, Shizuoka, Japan; 41 Department of Cardiovascular Surgery, Japanese Red Cross Wakayama Medical Center, Wakayama, Japan; 42 Department of Cardiovascular Surgery, National Hospital Organization Kyoto Medical Center, Kyoto, Japan; 43 Department of Cardiovascular Surgery, Cardiovascular Center, The Tazuke Kofukai Medical Research Institute, Kitano Hospital, Osaka, Japan; 44 Department of Cardiovascular Surgery, Hyogo Prefectural Amagasaki General Medical Center, Amagasaki, Japan; 45 Department of Cardiovascular Surgery, Rakuwakai Otowa Hospital, Kyoto, Japan; 46 Department of Cardiovascular Surgery, Shiga Medical Center for Adults, Moriyama, Japan; 47 Department of Cardiovascular Surgery, Hamamatsu Rosai Hospital, Hamamatsu, Japan; 48 Department of Cardiovascular Surgery, Japanese Redn Cross Otsu Hospital, Otsu, Japan

## Abstract

Prognostic impact of anemia complicating severe aortic stenosis (AS) remains unclear. We assessed the impact of anemia on cardiovascular and bleeding outcomes in 3403 patients enrolled in the CURRENT AS registry. 835 patients (25%) had mild (hemoglobin 11.0–12.9 g/dl for men/11.0–11.9 g/dl for women) and 1282 patients (38%) had moderate/severe anemia (Hb ≤ 10.9 g/dl) at diagnosis of severe AS. Mild and moderate/severe anemia were associated with significantly increased risks relative to no anemia (hemoglobin ≥13.0 g/dl for men/≥12.0 g/dl for women) for the primary outcome measure (aortic valve-related death or heart failure hospitalization) in the entire population [hazard ratio (HR): 1.30; 95% confidence interval (CI): 1.07–1.57 and HR: 1.56; 95%CI: 1.31–1.87, respectively] and in the conservative management stratum (HR: 1.73; 95%CI: 1.40–2.13 and HR: 2.05; 95%CI: 1.69–2.47, respectively). Even in the initial aortic valve replacement stratum, moderate/severe anemia was associated with significantly increased risk for the primary outcome measure (HR: 2.12; 95%CI: 1.44–3.11). Moreover, moderate/severe anemia was associated with significantly increased risk for major bleeding while under conservative management (HR: 1.93; 95%CI: 1.21–3.06). These results warrant further study to explore whether better management of anemia would lead to improvement of clinical outcomes.

## Introduction

Aortic stenosis (AS) is the most common valvular disease with poor prognosis and complex pathophysiology^1,2^. The majority of patients with AS are elderly with multiple co-morbidities causing poor functional status and prognosis^3^. Aortic valve replacement (AVR), either surgical or via a transcatheter approach, is the only therapeutic option in patients with severe AS, while there is no proven medical therapy for improving the prognosis of severe AS^4^. The identification of modifiable comorbidities might lead to improvement in outcomes for patients with severe AS.

Anemia is common in the elderly population and is potentially treatable^5^. Patients with severe AS are particularly susceptible to anemia, because they frequently are on antiplatelet and/or anticoagulant treatment and often suffer from acquired coagulopathy (von Willebrand syndrome type 2A), leading to an increased risk of bleeding^6,7^. Because tissue oxygen supply is limited due to decreased cardiac output, the concurrent presence of even a mild degree of anemia may harmfully affect the disease course of severe AS. Importantly, pre-existing anemia at the diagnosis of severe AS might be associated with a higher risk of future bleeding events, because anemia could be the result from a longstanding bleeding tendency.

Several recent studies have focused on the relationship between anemia and severe AS^8–11^. However, most of those studies included only patients who underwent transcatheter aortic valve implantation (TAVI), and anemia was diagnosed during the periprocedural period. Given a considerable number of patients with severe AS patients who are under medical management or a watchful waiting strategy in daily clinical practice^12^, it would be pertinent to evaluate the prognostic impact of anemia present at the time of severe AS diagnosis.

Therefore, we comprehensively evaluated the characteristics of severe AS patients with anemia enrolled consecutively in a large Japanese multicenter registry and assessed the impact of anemia on cardiovascular as well as bleeding outcomes.

## Methods

### Study Population

The study design and primary results of the CURRENT AS (Contemporary Outcomes After Surgery and Medical Treatment in Patients with Severe Aortic Stenosis) registry have been previously reported^13^. Briefly, the CURRENT AS registry is a retrospective, multicenter registry that enrolled 3815 consecutive patients who met the definition of severe AS (i.e. peak aortic jet velocity [Vmax] > 4.0 m/s, mean aortic pressure gradient [PG] > 40 mmHg, or aortic valve area [AVA] < 1.0 cm^2^) for the first time between January 2003 and December 2011 at 27 centers in Japan. The institutional review board or ethics committee at all 27 participating centers approved the study protocol. Written informed consent was waived by all review boards/ethics committees, because we retrospectively gathered the data obtained in the routine clinical practice, and no patient refused to participate in the study when contacted for follow-up. The study was performed in accordance with the relevant guidelines and regulations.

The current study population consisted of 3403 patients with severe AS after excluding 412 patients whose baseline hemoglobin (Hb) values were not available. The study patients were categorized into 3 groups based on the baseline Hb values according to the standard World Health Organization classification of anemia: no anemia (Hb ≥ 13.0 g/dl for men, and ≥12.0 g/dl for women), mild anemia (Hb 11.0–12.9 g/dl for men, and 11.0–11.9 g/dl for women), and moderate/severe anemia (Hb < 10.9 g/dl)^14^. The median time between the index echocardiography and baseline blood test was 1 day (interquartile range [IQR], 0–10 days).

Follow-up was commenced on the day of the index echocardiography. Follow-up information was collected primarily through review of hospital charts, and additional information was collected from patients, relatives and/or referring physicians via a mailed questionnaire regarding survival, symptoms and subsequent hospitalizations.

### Definitions of the Clinical Outcome Measures

The primary outcome measure in the present analysis was the AS-related clinical outcome, namely a composite of aortic valve-related death and heart failure (HF) hospitalization. The secondary outcome measures included the individual components of the primary outcome measure as well as all-cause death, cardiovascular death, sudden death, and non-cardiovascular death. Aortic valve-related death included aortic valve procedure death, sudden death, and death due to HF possibly related to AS. Causes of death were defined according to the Valve Academic Research Consortium (VARC) criteria^15,16^. HF hospitalization was defined as hospitalization due to deteriorating HF that required intravenous drug therapy. The severity of bleeding events was classified by using Bleeding Academic Research Consortium (BARC) types in accordance with the VARC-2 criteria; major and life-threatening/disabling bleeding in the present study was defined as BARC type 3, and type 5 (Supplementary Data)^16,17^. Other definitions of clinical events have been described previously^13^. A clinical event committee adjudicated all the clinical events (Supplementary Data).

### Statistical Analysis

We compared the baseline characteristics among the 3 groups categorized based on the status of anemia, and explored the independent factors associated with anemia. We also evaluated the prognostic impact of anemia, including stratified analyses according to the initial treatment strategies such as initial AVR and conservative strategies.

Categorical variables are presented as numbers and percentages; these were compared with the chi-square test or Fisher’s exact test. Continuous variables are expressed as the mean and standard deviation or median and IQR. For comparisons across the 3 groups of anemia status, we used analysis of variance or Kruskal-Wallis test.

We explored the factors associated with the presence of mild or moderate/severe anemia by the univariate and multivariable logistic regression models. We simultaneously included the 17 clinically relevant variables listed in Supplementary Table S1 as well as anemia (both mild and moderate/severe) in the model. Continuous variables were dichotomized according to the median value or a clinically meaningful reference value.

The cumulative incidences of the clinical events were estimated by the Kaplan–Meier method, and differences across the 3 groups were assessed with the log-rank test. The risks of mild anemia and moderate/severe anemia, respectively, relative to no anemia (reference) for the primary and secondary outcome measures were estimated by the Cox proportional hazard models and expressed as hazard ratios (HRs) and their 95% confidence intervals (CIs). We used the dummy code for mild anemia and moderate/severe anemia to estimate the HRs relative to no anemia in the models. Consistent with our previous report, the 22 clinically relevant factors listed in Supplementary Tables S2 and S3 were included as the risk-adjusting variables and the centers were incorporated as the stratification variable in the multivariable Cox proportional hazard models in the entire cohort. Except for age, continuous variables were dichotomized by median or clinically meaningful reference values. We also performed subgroup analyses stratified by clinically relevant factors, such as the initial treatment strategy (initial AVR and conservative), age, symptomatic status, severity of AS, left ventricular systolic function, and renal function. In the subgroup analysis stratified by the initial therapeutic strategy, we constructed parsimonious models with the 6 clinically most relevant risk-adjusting variables listed in Supplementary Tables S2 and S3, because of the small number of patients with outcome. Other than that, the same 22 risk-adjusting variables used in the entire cohort were included in the multivariable Cox proportional hazard models in the subgroup analyses. For those outcome measures with small numbers of patients with events such as sudden death and non-cardiovascular death, multivariable analysis was not performed. We conducted the interaction analyses using a Cox model containing interactive variables (a subgroup term, anemia term and anemia-by-subgroup term) and risk-adjusting variables. Global P for anemia-by-subgroup term was calculated as P for interaction^18^. For the evaluation of bleeding events, we censored patients at the time of AVR/TAVI, because we did not collect data on bleeding and transfusion in the perioperative period. Therefore, we estimated the incidences of the bleeding events specifically while under medical therapy. In the adjusted analyses on the risks of each anemia group for major bleeding events, the same 22 factors as those included in the main analyses were incorporated into multivariable Cox proportional hazard models as the risk adjusting variables and the centers were incorporated as the stratification variable.

As a sensitivity analysis, the risks of the mild and moderate/severe anemia relative to the no anemia for the primary outcome measure and bleeding events were estimated by the Cox proportional hazard models accounting for the competing risk of AVR/TAVI by using the Gray method^19^.

All statistical analyses were performed with the statistical software program JMP 10.0.0 (SAS Institute Inc., Cary, NC, USA) and SAS 9.4 (SAS Institute Inc., Cary, NC, USA). All reported P values are two-tailed. P values < 0.05 were considered statistically significant.

## Results

### Baseline Characteristics According to the Severity of Anemia

**Figure 1:**
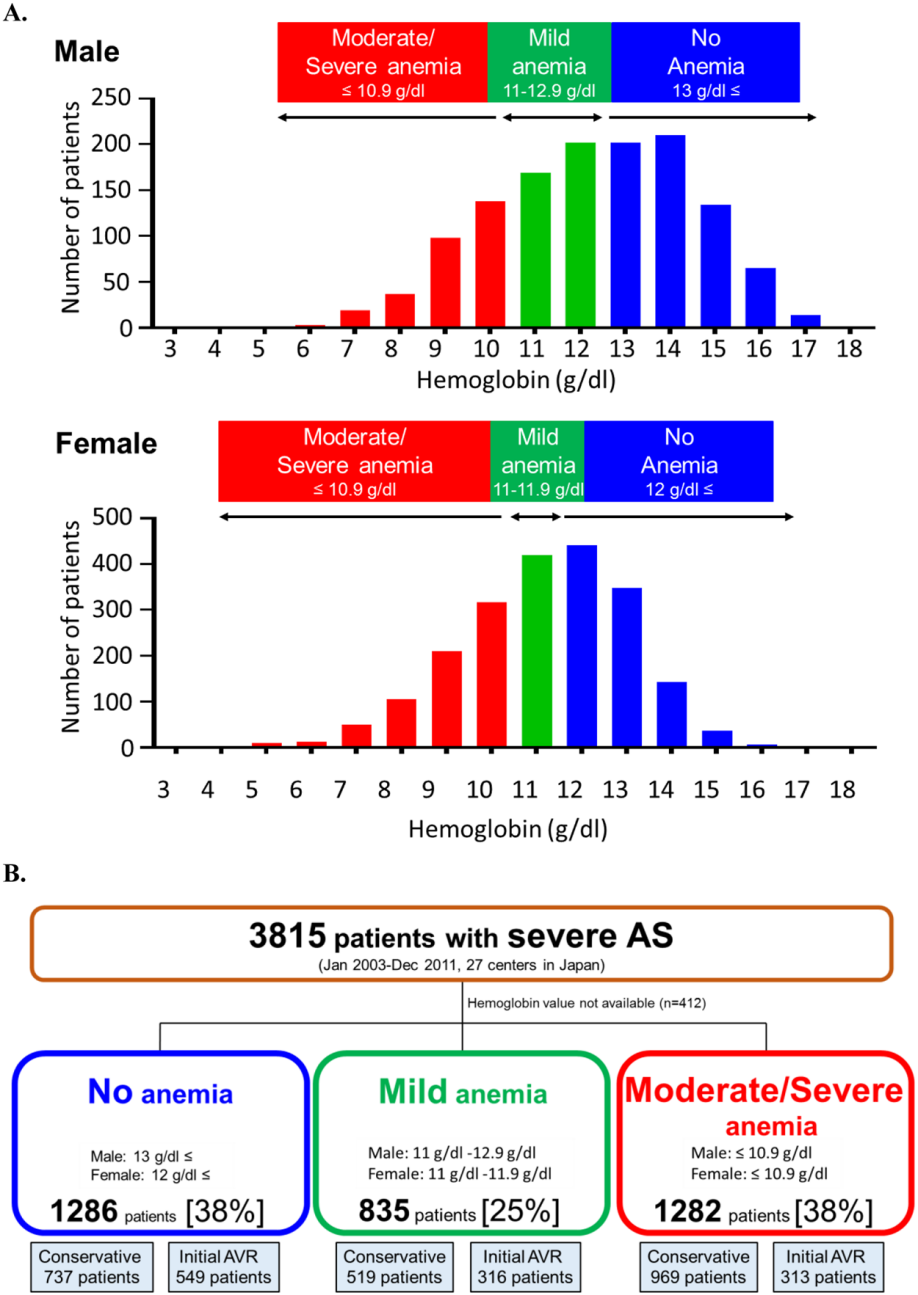
(**A**) Histograms of hemoglobin levels. (**B**) Study flowchart AS = aortic stenosis, AVR = aortic valve replacement.

A large proportion of patients in the present study had anemia; there were 1286 patients (38%) without anemia, and 2117 patients (62%) with anemia, of whom 835 (25%) had mild anemia and 1282 (38%) had moderate/severe anemia (Fig. 1A,B). Median Hb values were 13.4 (IQR: 12.7–14.2) g/dl, 11.6 (11.3–11.9) g/dl, and 9.7 (8.7–10.4) g/dl in the no anemia, mild anemia, and moderate/severe anemia groups, respectively (P < 0.001). Baseline characteristics differed significantly across the 3 groups (Table 1). Overall, patients with moderate/severe anemia were older, more likely to be female, had lower body mass index (BMI), were less likely to have dyslipidemia and more often had a history of HF or malignancy than those with no or mild anemia (Table 1). Patients in the 2 anemia groups more often had a history of percutaneous coronary intervention or coronary artery bypass graft and history of aortic/peripheral vascular diseases than those without anemia. Serum creatinine, brain-derived natriuretic peptide (BNP), C-reactive protein (CRP) and surgical risk scores were higher with increasing severity of anemia (Table 1). Regarding the echocardiographic parameters, compared with patients with no or mild anemia, those with moderate/severe anemia had lower Vmax, smaller AVA, lower left ventricular ejection fraction, thinner wall thickness and greater tricuspid regurgitation PG (Table 1). Proportion of patients with low gradient severe AS (Vmax ≤ 4 m/s and mean aortic PG ≤ 40 mmHg, but AVA < 1.0 cm^2^) was higher in the patients with moderate/severe anemia as compared with those with no or mild anemia. An initial AVR strategy was selected in 1178 patients (35% of the cohort), of whom 1156 (98.1%) actually underwent surgical AVR (n = 1145) or TAVI (n = 11) at a median interval of 36 (IQR: 16–60) days from the index echocardiography (Table 1). Among the remaining 2225 patients for whom the conservative strategy was initially selected, 451 (20.3%) eventually underwent surgical AVR (n = 429) or TAVI (n = 23) at a median interval of 756 (IQR: 270–1268) days from the index echocardiography (Table 1). Initial AVR strategy was selected less often, and AVR or TAVI was performed less often as the anemia severity increased (Table 1). Further detailed data on baseline characteristics were provided in Supplemental Table S2.

**Table 1:** Baseline Characteristics According to the Status of Anemia.

Variables	No anemia Hb ≥ 13.0 g/dl for men, and ≥12.0 g/dl for women	Mild anemia Hb 11.0–12.9 g/dl for men, and 11.0–11.9 g/dl for women	Moderate/Severe anemia Hb ≤ 10.9 g/dl	P value
	N = 1286	N = 835	N = 1282	
**Clinical characteristics**				
Age, y	74.5 ± 9.9	78.0 ± 8.7	81.4 ± 8.7	<0.001
Men	524 (41)	399 (48)	371 (29)	<0.001
BMI, kg/m^2^	22.7 ± 3.7	22.0 ± 3.7	20.6 ± 3.6	<0.001
Hypertension	879 (68)	598 (72)	923 (72)	0.09
Current smoking	87 (7)	51 (6)	37 (3)	<0.001
Dyslipidemia	514 (40)	308 (37)	372 (29)	<0.001
Diabetes mellitus	296 (23)	209 (25)	323 (25)	0.4
Coronary artery disease	341 (27)	304 (36)	398 (31)	<0.001
Prior PCI	126 (10)	132 (16)	189 (15)	<0.001
Prior CABG	38 (3)	58 (7)	72 (6)	<0.001
Prior myocardial infarction	73 (6)	78 (9)	132 (10)	<0.001
Prior HF	158 (12)	119 (14)	314 (24)	<0.001
Aortic/peripheral vascular disease	156 (12)	152 (18)	227 (18)	<0.001
Serum creatinine, mg/dl	0.8 (0.6–1.0)	0.9 (0.7–1.2)	1.1 (0.8–2.3)	<0.001
Hemoglobin, g/dl	13.4 (12.7–14.2)	11.6 (11.3–11.9)	9.7 (8.7–10.4)	<0.001
BNP, pg/ml	143 (57–432)	216 (97–615)	554 (202–1357)	<0.001
CRP, mg/dl	0.13 (0.06–0.36)	0.2 (0.08–0.64)	0.43 (0.1–2.2)	<0.001
Malignancy	145 (11)	114 (14)	216 (17)	<0.001
Chronic lung disease	159 (12)	88 (11)	119 (9)	0.04
Logistic EuroSCORE, %	7.0 (4.2–12.0)	9.4 (6.2–16.0)	14.2 (9.0–23.0)	<0.001
EuroSCORE II,%	1.9 (1.2–3.5)	2.8 (1.7–4.5)	4.1 (2.7–6.8)	<0.001
STS score (PROM), %	2.5 (1.6–4.0)	3.8 (2.4–5.9)	6.1 (3.7–1.0)	<0.001
**Echocardiographic variables**				
Vmax, m/s	4.2 ± 0.9	4.2 ± 0.9	4.1 ± 0.9	0.01
Mean aortic PG, mmHg	42 ± 20	42 ± 21	41 ± 20	0.09
AVA (equation of continuity), cm2	0.73 ± 0.18	0.72 ± 0.18	0.69 ± 0.19	<0.001
Low gradient AS (Vmax ≤ 4 m/s and mean	537 (42)	356 (43)	597 (47)	0.04
aortic PG ≤ 40 mmHg, but AVA < 1.0 cm^2^)				
LVEF, %	64 ± 13	63 ± 13	61 ± 14	<0.001
IVST in diastole, mm	11.5 ± 2.4	11.5 ± 2.3	11.2 ± 2.2	0.002
PWT in diastole, mm	11.1 ± 2.0	11.1 ± 2.2	10.9 ± 2.0	0.04
TR pressure gradient ≥40 mm Hg	158 (12)	117 (14)	292 (23)	<0.001
**Therapeutic strategy**				
Initial AVR	549 (43)	316 (38)	313 (24)	<0.001
Conservative	737 (57)	519 (62)	969 (76)	<0.001

Values are mean ± SD, median (interquartile range), or number (%). The values of CRP and BNP were obtained in 2914 (76%) and 1801 (47%) patients, respectively. Further detailed data on baseline characteristics were provided in Supplemental Table S2.

AS = aortic stenosis; AVA = aortic valve area; AVR = aortic valve replacement; BMI = body mass index; BNP = brain-derived natriuretic peptide; CABG = coronary artery bypass grafting; CRP = C-reactive protein; Hb = hemoglobin; HF = heart failure; IVST = interventricular septum thickness, LVEF = left ventricular ejection fraction; PCI = percutaneous coronary intervention; PG = pressure gradient; PROM = predicted risk of mortality; PWT = posterior wall thickness; STS = Society of Thoracic Surgeons; TR = tricuspid regurgitation; Vmax = peak aortic jet velocity.

### Factors Associated with Anemia

Variables independently associated with anemia included older age, female gender, lower BMI, coronary artery disease and aortic/peripheral disease, renal failure, prior HF, malignancy, liver cirrhosis, and higher tricuspid regurgitation PG (≥40 mmHg) (Supplementary Table S1).

### Primary Outcome Measure According to the Severity of Anemia: Entire Cohort

**Figure 2:**
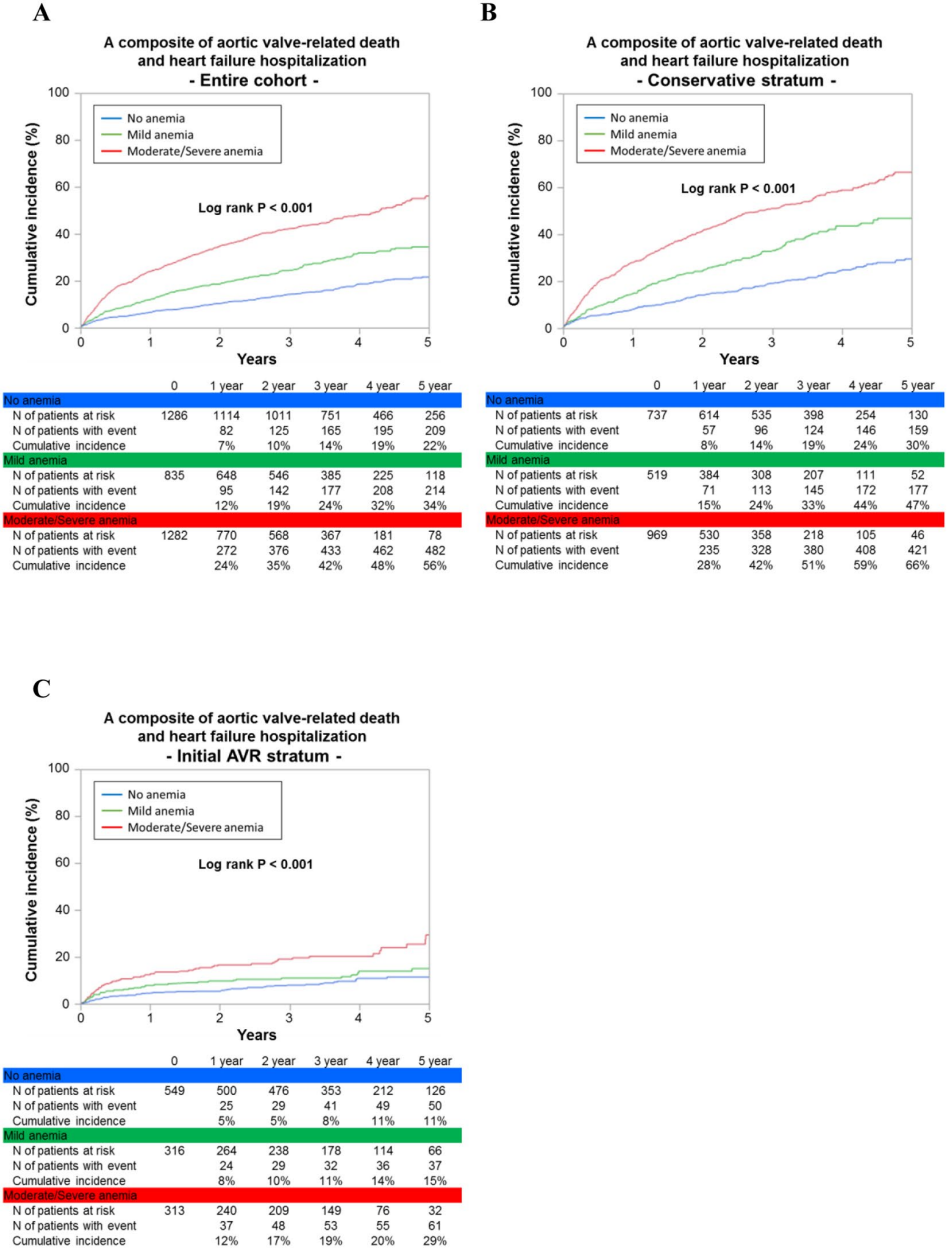
Kaplan–Meier curves for the primary outcome measure according to the severity of anemia. (**A**) Entire cohort. (**B**) Conservative stratum. (**C**) Initial AVR stratum. The primary outcome measure was defined as a composite of aortic valve-related death of heart failure hospitalization. Severity of anemia was classified as no anemia (Hb ≥ 13.0 g/dl for men, and≥12.0 g/dl for women), mild anemia (Hb 11.0–12.9 g/dl for men, and 11.0–11.9 g/dl for women), and moderate/severe anemia (Hb ≤ 10.9 g/dl). AVR = aortic valve replacement, and Hb = hemoglobin.

The cumulative 5-year incidence of the primary outcome measure (a composite of aortic valve-related death and HF hospitalization) increased with increasing severity of anemia (22%, 34%, and 56% in the no, mild, and moderate/severe anemia groups, respectively; P < 0.001) (Fig. 2A). Even after adjusting for potential confounders, the excess risk of the mild and moderate/severe anemia groups relative to the no anemia group for the primary outcome measure remained significant (HR: 1.30; 95% CI: 1.07–1.57; P = 0.008, and HR: 1.56; 95% CI: 1.31–1.87; P < 0.001, respectively) (Table 2). When we censored the patients at the time of AVR/TAVI to evaluate the impact of anemia while under conservative management, the excess risks of the 2 anemia groups relative to the no anemia group for the primary outcome measure remained significant (adjusted HR: 1.46; 95% CI: 1.17–1.82; P < 0.001, and adjusted HR: 1.69; 95% CI: 1.38–2.07; P < 0.001, respectively).

**Table 2:** Crude and Adjusted Effects of Anemia for Clinical Outcomes.

	No anemia	Mild anemia versus No anemia					Moderate/Severe anemia versus No anemia				
	N of patients with event (Cumulative 5-year incidence, %)	N of patients with event (Cumulative 5-year incidence, %)	Unadjusted HR (95% CI)	P Value	Adjusted HR (95% CI)	P Value	N of patients with event (Cumulative 5-year incidence, %)	Unadjusted HR (95% CI)	P Value	Adjusted HR (95% CI)	P Value
**Entire Cohort (N = 3403)**											
**Primary outcome measure**											
Aortic valve-related death and HF hospitalization	209 (22)	214 (34)	1.71 (1.43–2.05)	<0.001	1.30 (1.07–1.57)	0.008	482 (56)	3.31 (2.83–3.87)	<0.001	1.56 (1.31–1.87)	<0.001
**Secondary outcome measures**											
Aortic valve-related death	101 (11)	119 (21)	1.93 (1.50–2.48)	<0.001	1.29 (0.99–1.67)	0.06	293 (36)	4.03 (3.25–4.99)	<0.001	1.45 (1.14–1.85)	0.003
HF hospitalization	158 (18)	158 (28)	1.70 (1.38–2.10)	<0.001	1.39 (1.12–1.73)	0.003	362 (48)	3.44 (2.88–4.12)	<0.001	1.79 (1.46–2.20)	<0.001
All-cause death	263 (26)	282 (43)	1.85 (1.58–2.18)	<0.001	1.20 (1.02–1.42)	0.03	677 (65)	3.73 (3.26–4.28)	<0.001	1.62 (1.39–1.89)	<0.001
Cardiovascular death	163 (17)	189 (31)	1.92 (1.57–2.35)	<0.001	1.24 (1.01–1.52)	0.04	448 (50)	3.92 (3.31–4.65)	<0.001	1.52 (1.25–1.84)	<0.001
Sudden death	34 (3)	40 (7)	1.79 (1.18–2.72)	0.007	1.003 (0.64–1.57)	1.0	83 (14)	2.95 (2.05–4.26)	<0.001	1.07 (0.69–1.64)	0.8
Non-cardiovascular death	100 (12)	93 (17)	1.74 (1.33–2.27)	<0.001	1.13 (0.85–1.49)	0.4	229 (29)	3.40 (2.71–4.28)	<0.001	1.83 (1.41–2.38)	<0.001
**Conservative Stratum (N = 2225)**											
**Primary outcome measure**											
Aortic valve-related death and HF hospitalization	159 (30)	177 (47)	1.79 (1.46–2.20)	<0.001	1.73 (1.40–2.13)	<0.001	421 (66)	3.13 (2.63–3.74)	<0.001	2.05 (1.69–2.47)	<0.001
**Secondary outcome measures**											
Aortic valve-related death	80 (16)	105 (31)	2.04 (1.55–2.69)	<0.001	1.90 (1.44–2.51)	<0.001	261 (45)	3.63 (2.89–4.62)	<0.001	2.14 (1.66–2.76)	<0.001
HF hospitalization	129 (26)	134 (39)	1.71 (1.36–2.16)	<0.001	1.63 (1.28–2.06)	<0.001	329 (59)	3.18 (2.62–3.88)	<0.001	2.05 (1.66–2.54)	<0.001
All-cause death	207 (36)	224 (54)	1.79 (1.50–2.15)	<0.001	1.52 (1.26–1.83)	<0.001	586 (72)	3.25 (2.8–3.8)	<0.001	2.09 (1.77–2.46)	<0.001
Cardiovascular death	125 (23)	155 (41)	1.94 (1.55–2.43)	<0.001	1.76 (1.40–2.22)	<0.001	387 (58)	3.46 (2.86–4.19)	<0.001	2.19 (1.79–2.69)	<0.001
Sudden death	25 (4)	36 (10)	2.01 (1.26–3.21)	0.003	N/A		73 (15)	2.7 (1.8–4.19)	<0.001	N/A	
Non-cardiovascular death	82 (17)	69 (21)	1.54 (1.13–2.10)	0.006	N/A		199 (33)	2.92 (2.27–3.78)	<0.001	N/A	
**Initial AVR Stratum (N = 1178)**											
**Primary outcome measure**											
Aortic valve-related death and HF hospitalization	50 (11)	37 (15)	1.36 (0.90–2.02)	0.1	1.24 (0.82–1.88)	0.3	61 (29)	2.47 (1.73–3.54)	<0.001	2.12 (1.44–3.11)	<0.001
**Secondary outcome measures**											
Aortic valve-related death	21 (4)	14 (5)	1.21 (0.6–2.4)	0.6	1.16 (0.58–2.32)	0.7	32 (11)	3.04 (1.77–5.34)	<0.001	2.94 (1.64–5.26)	<0.001
HF hospitalization	29 (7)	24 (11)	1.5 (0.91–2.45)	0.1	1.30 (0.77–2.19)	0.3	33 (22)	2.33 (1.46–3.71)	0.002	1.79 (1.08–2.97)	0.02
All-cause death	56 (13)	58 (25)	1.89 (1.34–2.67)	<0.001	1.88 (1.31–2.69)	<0.001	91 (42)	3.66 (2.69–5.02)	<0.001	3.62 (2.57–5.08)	<0.001
Cardiovascular death	38 (9)	34 (15)	1.66 (1.06–2.58)	0.03	1.70 (1.07–2.68)	0.02	61 (29)	3.81 (2.61–5.53)	<0.001	3.94 (2.60–5.95)	<0.001
Sudden death	9 (2)	4 (2)	0.86 (0.27–2.38)	0.8	N/A		11 (10)	2.37 (1.01–5.57)	0.03	N/A	
Non-cardiovascular death	18 (5)	24 (12)	2.34 (1.34–4.12)	0.005	N/A		30 (16)	3.36 (1.97–5.83)	<0.001	N/A	

AVR = aortic valve replacement; CI = confidence interval; HF = heart failure; HR = hazard ratio.

In the subgroup analyses, there were no significant interactions between the subgroup factors and the effect of anemia on the primary outcome measure except for the subgroups stratified by renal function (Supplementary Fig. S2).

### Primary Outcomes Measure According to the Severity of Anemia Stratified by Initial Therapeutic Strategy

In the conservative stratum, the cumulative 5-year incidence of AVR or TAVI decreased with increasing severity of anemia (43%, 37%, and 25%, respectively, P < 0.001), whereas in the initial AVR stratum, the vast majority of patients underwent AVR or TAVI regardless of the severity of anemia (Supplementary Fig. S3A,B). Regardless of the initial treatment strategies (initial AVR and conservative), the effects of anemia severity for the primary outcome measure were generally in the same direction as those in the entire cohort with no positive interaction between anemia severity and the initial therapeutic strategies (interaction P = 0.2) (Table 2), although the outcomes of each anemia group were remarkably better in the AVR than in the conservative stratum (Fig. 2B,C).

### Secondary Outcome Measures According to the Severity of Anemia

The effects of the severity of anemia for the secondary outcome measures such as aortic valve-related death, HF hospitalization, all-cause death and cardiovascular death were generally in the same direction as for the primary outcome measure in the entire cohort, and in the conservative stratum (Table 2, and Supplementary Fig. S1). In the initial AVR stratum, moderate/severe anemia as compared with no anemia was associated with significantly higher risk for all the secondary outcome measures, whereas mild anemia as compared with no anemia was associated with significantly higher risk only for all-cause death, cardiovascular death and non-cardiovascular death (Table 2). There were no significant interactions between the initial therapeutic strategies and the effect of anemia on the secondary outcome measures (interaction P = 0.08, 0.7, 0.2 and 0.06 for aortic valve-related death, HF hospitalization, all-cause death and cardiovascular death, respectively).

### Bleeding Events Under Conservative Management

**Figure 3:**
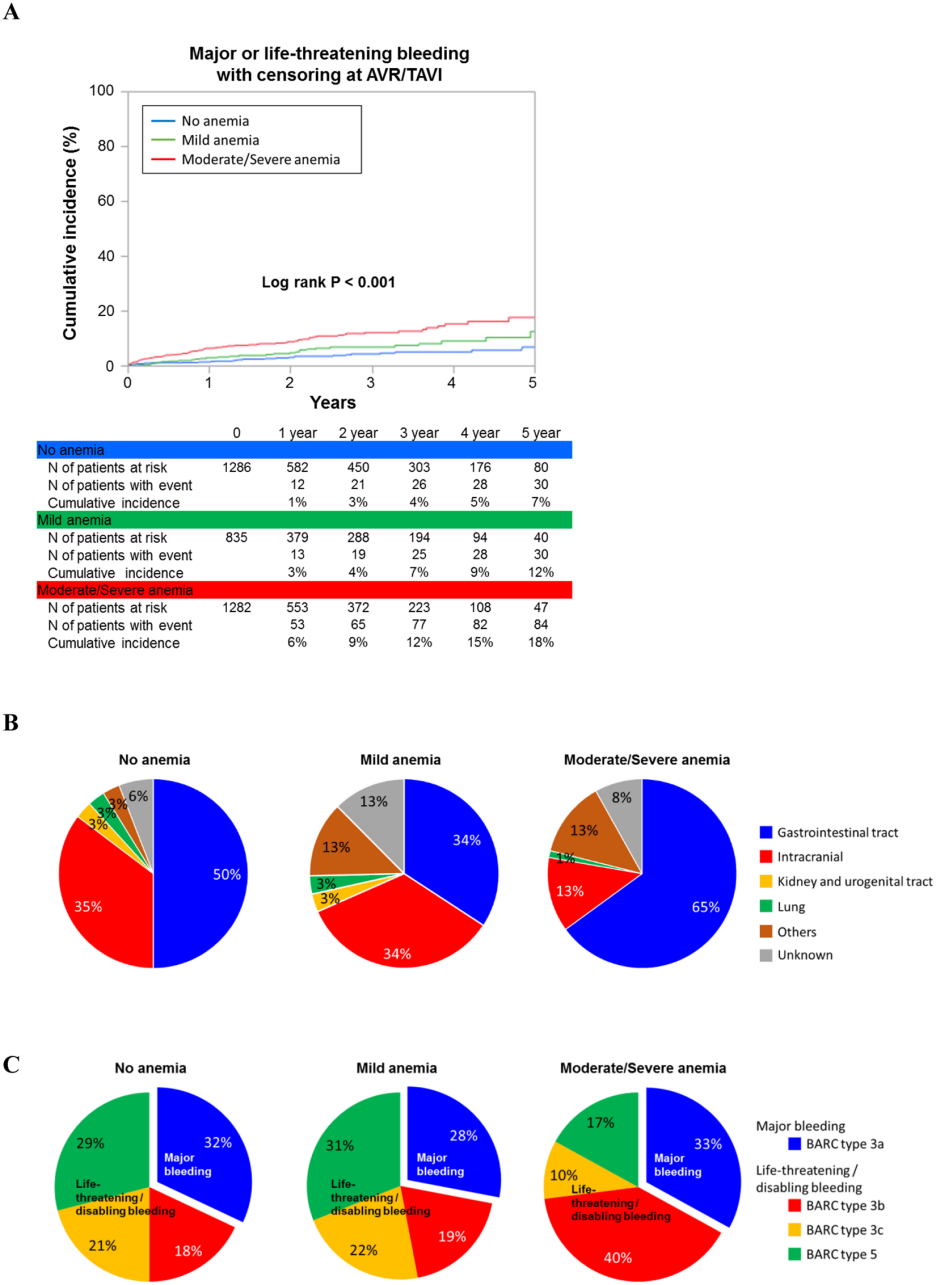
Relationship between anemia and bleeding events under conservative management. (**A**) Kaplan–Meier curves for major or life-threatening bleeding events under conservative management in the entire cohort. (**B**,**C**) Sites (**B**) and severity (**C**) of bleeding under conservative management in the entire cohort of bleeding. Cumulative incidence of major or life-threatening/disabling bleeding events under conservative management was estimated by the Kaplan-Meier method with censoring at AVR/TAVI. AVR = aortic valve replacement, BARC = Bleeding Academic Research Consortium, and TAVI = transcatheter aortic valve implantation.

In the entire cohort, 152 (4.5%) patients had major or life-threatening bleeding events while under conservative management. The cumulative 5-year incidence of bleeding events was incrementally higher with the increasing severity of anemia (7%, 12%, and 18%, respectively, P < 0.001) (Fig. 3A). After adjusting for the potential confounders, the excess risk of the moderate/severe anemia group relative to the no anemia group remained highly significant (HR: 1.93; 95%CI: 1.21–3.06; P = 0.005), whereas no significant increased risk was observed for the mild anemia group relative to the no anemia group (adjusted HR: 1.15, 95%CI: 0.69–1.91, P = 0.6). Gastrointestinal (55%) and intracranial bleeding (22%) were the two main bleeding sites (Fig. 3B). One-third of the bleeding events was major bleeding (BARC type 3a), whereas two-thirds of the bleeding events were life-threatening or disabling bleeding (BARC types 3b, 3c and 5) (Fig. 3C).

### Sensitivity analysis for the excess risk of anemia accounting for the competing risk of AVR/TAVI

Sensitivity analysis confirmed that even when the competing risk of AVR/TAVI was accounted for, the excess risks relative to the no anemia group for the primary outcome measure remained significant in both the mild (unadjusted HR: 1.75; 95% CI: 1.43–2.15; P < 0.001, adjusted HR: 1.43; 95% CI: 1.15–1.77; P = 0.001) and the moderate/severe anemia group (unadjusted HR: 3.45; 95% CI: 2.90–4.10; P < 0.001, adjusted HR: 1.60; 95% CI: 1.30–1.96; P < 0.001). Likewise, the adjusted excess risk of the moderate/severe anemia group relative to the no anemia group for the major or life-threatening bleeding events remained highly significant (unadjusted HR: 3.33; 95% CI: 2.24–4.94; P < 0.001, adjusted HR: 1.92; 95% CI: 1.24–2.99; P = 0.004) even when the competing risk of AVR/TAVI was accounted for, whereas no significant excess risk relative to the no anemia group was observed in the mild anemia group (unadjusted HR: 1.57; 95%CI: 0.97–2.53, P = 0.07, adjusted HR: 1.92; 95% CI: 1.24–2.99; P = 0.004).

## Discussion

In a large cohort of patients with severe AS, we found that more than 60% of patients had anemia at the time of severe AS diagnosis. Moderate/severe anemia was associated with extremely worse prognosis with increased risk for AS-related adverse events regardless of the therapeutic strategy. Even a mild degree of anemia was associated with significantly worse prognosis in the entire population and in the patients who were medically managed. Furthermore, moderate/severe anemia was associated with increased risk of major or life-threatening bleeding while under medical therapy.

Previous reports are limited for the prognostic impact of Hb levels at severe AS diagnosis. In one study exploring the relationship between baseline anemia and prognosis in 856 AS patients, the prevalence of anemia increased with increasing severity of AS, and anemia was independently associated with increased all-cause mortality while under medical therapy, but not after AVR surgery^9^. Of note, the patients included in that study were much younger (mean age, 71 years) than those in our study and had less severe AS, with more than 50% of their patients having moderate AS. This may be related to a much lower prevalence of anemia in their cohort (32%) as compared with ours (63%). In other cohorts including the patients who underwent TAVI, the prevalence of preoperative anemia was 45–64%^8,10,20,21^. We found several predisposing factors to anemia such as older age, low BMI, a history of HF, coronary artery disease and aortic/peripheral disease, renal failure and malignancy. The results may reflect the growing prevalence of severe AS in the elderly population with multiple comorbidities^3^, and all these factors may synergistically contribute to the extremely poor prognosis in severe AS patients associated with anemia. Particularly, end-stage renal function deleteriously affects the prognosis of severe AS, as reported in our previous study^22^. Nevertheless, even after careful adjustment for a broad array of baseline characteristics including renal function, we still found anemia to be a strong indicator of poor prognosis. AVR/TAVI strategy was selected less often in patients with higher-grade anemia, which might have increased the rate of clinical events in the entire cohort. However, even in the initial AVR stratum in which more than 98% of the patients underwent AVR, the cumulative 5-year incidence of the primary outcome measure still was incrementally higher with increasing severity of anemia. Furthermore, even a mild degree of anemia was associated with significantly worse outcomes; its deleterious effect was prominent in those patients with advanced age, without symptoms, without ‘very severe’ AS (Vmax < 5 m/s) and with preserved left ventricular systolic function (ejection fraction ≥50%). Notably, these factors might predispose to the selection of conservative strategy rather than initial AVR strategy^13,23,24^. Importantly, in contrast to the previous reports from TAVI cohorts, our study enrolled consecutive patients with severe AS, and therefore, included substantial proportion of patients who were managed conservatively^8,10,11^. Negative prognostic impact of anemia was more prominent in patients with a conservative strategy than in those with an initial AVR strategy. Given these results, together with lack of effective medical management for severe AS^4,23,25^, anemia might be an important target of medical management in patients with severe AS. For example, iron therapy, which has been proven for improving the functional status of chronic HF patients, might be a viable therapeutic option for patients with severe AS, which should be evaluated in prospective studies^26,27^.

We found that the patients with baseline anemia had an elevated risk of major or life-threatening bleeding events as compared with those without anemia. Similarly, Philippe *et al*. reported that the presence of low Hb levels at baseline was significantly associated with major bleeding complications within 30 days of surgical AVR^28^. The presence of anemia at severe AS diagnosis could be the result from longstanding bleeding tendency, possibly due to the continuous prescription of antithrombotic drugs, or von Willebrand syndrome type 2A^7,29^. Importantly, the presence of anemia at the diagnosis of severe AS often might be regarded as ‘not severe’, especially in elderly patients. However, given the highly significant association between the presence of anemia and the extremely poor prognosis demonstrated in our study, we might have need to pay more attention to anemia in patients with severe AS.

### Limitations

This study had several limitations. First, anemia was evaluated only at baseline. Therefore, the subsequent change in Hb and its relationship with the prognosis remained unclear. Second, the relationship between baseline anemia and the incidence of AVR/TAVI-related bleeding events remains unclear, because our study focused more on the bleeding events under conservative management rather than on procedure-related events. Third, to keep consistency with our previous reports, the same clinically relevant factors as in our previous reports were included as the risk-adjusting variables in the Cox proportional hazard models. However, this strategy might result in overfitting models particularly in the analyses for some secondary outcomes and bleeding events. Fourth, patients with anemia were more likely to be frail, have a history of HF, malignancy and coronary artery or aortic/peripheral disease than those without anemia. In addition, they were more likely to have higher BNP, CRP and surgical risk scores. Despite an extensive statistical adjustment for potential confounders obtained in our registry, we cannot deny the residual unmeasured confounders such as frailty^30^. Finally, it should be acknowledged that the CURRENT AS registry included mostly Asian patients and, hence, limits the generalizability of the study to mostly Asian patients with AS.

(Contemporary Outcomes After Surgery and Medical Treatment in Patients With Severe Aortic Stenosis Registry; UMIN000012140). https://upload.umin.ac.jp/cgi-open-bin/ctr_e/ctr_view.cgi?recptno=R000014041.

## Conclusions

Anemia is a common comorbidity in patients with severe AS and is associated with worse cardiovascular as well as bleeding outcomes. Further study should be warranted to explore whether better management of anemia would lead to improvement of clinical outcomes.

## Electronic supplementary material

Supplementary Information
