# Supplementary material for: Anemia in Patients with Severe Aortic Stenosis
Source: Sci Rep. 2019 Feb 13;9:1924. doi: 10.1038/s41598-018-36066-z (PMC6374463; doi:10.1038/s41598-018-36066-z)
Supplement: Supplementary file 1 — Supplementary Information [file 41598_2018_36066_MOESM1_ESM.pdf]

Supplementary Information

Anemia in Patients with Severe Aortic Stenosis

Kazuya Nagao, MD[1], Tomohiko Taniguchi, MD[2], Takeshi Morimoto, MD, MPH[3], Hiroki Shiomi, MD[2], Kenji Ando, MD[4], Norio Kanamori, MD[5], Koichiro Murata, MD[6], Takeshi Kitai, MD[7], Yuichi Kawase, MD[8], Chisato Izumi, MD[9], Makoto Miyake, MD[9], Hirokazu Mitsuoka, MD[10], Masashi Kato, MD[11], Yutaka Hirano, MD[12], Shintaro Matsuda, MD[2], Tsukasa Inada, MD[1], Tomoyuki Murakami, MD[13], Yasuyo Takeuchi, MD[14], Keiichiro Yamane, MD[15], Mamoru Toyofuku, MD[16], Mitsuru Ishii, MD[17], Eri Minamino-Muta, MD[18], Takao Kato, MD[18], Moriaki Inoko, MD[18], Tomoyuki Ikeda, MD[19], Akihiro Komasa, MD[20], Katsuhisa Ishii, MD[20], Kozo Hotta, MD[21], Nobuya Higashitani, MD[22], Yoshihiro Kato, MD[23], Yasutaka Inuzuka, MD[24], Chiyo Maeda, MD[25], Toshikazu Jinnai, MD[22], Yuko Morikami[26], MD, Naritatsu Saito, MD[2], Kenji Minatoya, MD[27], Takeshi Kimura, MD[2], on behalf of the CURRENT AS Registry Investigators

Definitions of the clinical events

Major bleeding was defined as overt bleeding plus a decrease in hemoglobin of 3 g/dl to <5 g/dl or bleeding requiring transfusion (BARC type 3a). Life-threatening/disabling bleeding events were defined as overt bleeding plus a decrease in hemoglobin ≥5 g/dl or bleeding requiring surgical intervention or intravenous vasoactive agents (BARC type 3b), bleeding in a critical organ such as intracranial, intraocular (BARC type 3c) or fatal bleeding (BARC type 5) [1, 2]. Death was regarded as cardiovascular in origin unless obvious non-cardiovascular causes could be identified. Aortic valve (AV) procedure-related death was defined as any death during the hospitalization for surgical aortic valve replacement (AVR) or trans-catheter aortic valve implantation (TAVI). AV-related death included AV procedure-related death, sudden death, and death due to heart failure presumably related to aortic stenosis [3].

Supplemental Reference

1. Mehran R, Rao SV, Bhatt DL, Gibson CM, Caixeta A, Eikelboom J, Kaul S, Wiviott SD, Menon V, Nikolsky E, Serebruany V, Valgimigli M, Vranckx P, Taggart D, Sabik JF, Cutlip DE, Krucoff MW, Ohman EM, Steg PG, White H. Standardized bleeding

definitions for cardiovascular clinical trials: A consensus report from the bleeding academic research consortium. Circulation. 2011;123(23):2736-47.

2. Kappetein AP, Head SJ, Genereux P, Piazza N, van Mieghem NM, Blackstone EH, Brott TG, Cohen DJ, Cutlip DE, van Es GA, Hahn RT, Kirtane AJ, Krucoff MW, Kodali S, Mack MJ, Mehran R, Rodes-Cabau J, Vranckx P, Webb JG, Windecker S, Serruys PW, Leon MB. Updated standardized endpoint definitions for transcatheter aortic valve implantation: The valve academic research consortium-2 consensus document. J Am Coll Cardiol. 2012;60(15):1438-54

3. Taniguchi T, Morimoto T, Shiomi H, Ando K, Kanamori N, Murata K, Kitai T, Kawase Y, Izumi C, Miyake M, Mitsuoka H, Kato M, Hirano Y, Matsuda S, Nagao K, Inada T, Murakami T, Takeuchi Y, Yamane K, Toyofuku M, Ishii M, Minamino-Muta E, Kato T, Inoko M, Ikeda T, Komasa A, Ishii K, Hotta K, Higashitani N, Kato Y, Inuzuka Y, Maeda C, Jinnai T, Morikami Y, Sakata R, Kimura T, Investigators CAR. Initial surgical versus conservative strategies in patients with asymptomatic severe aortic stenosis. J Am Coll Cardiol. 2015;66(25):2827-38.

Supplemental Table S1. Univariate and Multivariable Logistic Regression Analyses for Factors Associated with Anemia

Variables	Univariate Analyses				Multivariable Analyses	
	Anemia		OR (95% CI)	P value	OR (95% CI)	P value
	Yes<br>N=2117	No<br>N=1286				
Age ≥80 years	1164 (55)	393 (31)	2.78 (2.40-3.21)	<0.001	2.27 (1.94-2.67)	<0.001
Women	1347 (64)	762 (59)	1.3 (1.04-1.39)	0.01	1.51 (1.26-1.80)	<0.001
BMI <22 kg/m^2	1432 (68)	634 (49)	2.15 (1.87-2.48)	<0.001	1.91 (1.64-2.24)	<0.001
Current smoking	88 (4)	87 (7)	0.6 (0.44-0.81)	0.001	0.9 (0.64-1.24)	0.5
Coronary artery disease	702 (33)	341 (27)	1.37 (1.18-1.60)	<0.001	1.32 (1.11-1.57)	0.002
Prior HF	433 (20)	158 (12)	1.84 (1.51-2.24)	<0.001	1.36 (1.09-1.70)	0.007

Prior symptomatic stroke	302 (14)	166 (13)	1.12 (0.92-1.38)	0.3	0.9 (0.73-1.14)	0.4
Atrial fibrillation or flutter	483 (23)	270 (21)	1.11 (0.94-1.32)	0.2	0.9 (0.71-1.05)	0.1
Malignancy	330 (16)	145 (11)	1.45 (1.18-1.79)	<0.001	1.44 (1.15-1.81)	0.002
Aortic/peripheral disease	379 (18)	156 (12)	1.58 (1.29-1.94)	<0.001	1.4 (1.11-1.73)	0.004
Liver cirrhosis	29 (1)	8 (1)	2.22 (1.06-5.22)	0.03	2.63 (1.19-6.48)	0.02
Chronic lung disease (moderate or severe)	41 (3)	64 (3)	0.95 (0.64-1.42)	0.8	0.9 (0.58-1.39)	0.6
Creatinine level above median (0.83 mg/dl)	1358 (73)	492 (27)	2.89 (2.50-3.33)	<0.001	3.06 (2.59-3.62)	<.0001
Vmax >5 m/s	402 (19)	261 (20)	0.9 (0.77-1.10)	0.4	1.09 (0.90-1.32)	0.4
LVEF <40 %	190 (9)	90 (7)	1.31 (1.01-1.71)	0.04	0.88 (0.66-1.18)	0.4

Any combined valvular disease (moderate or severe)	947 (45)	475 (37)	1.38 (1.20-1.59)	<0.001	1.07 (0.91-1.26)	0.4
TR pressure gradient ≥40 mm Hg	409 (19)	158 (12)	1.71 (1.40-2.09)	<0.001	1.34 (1.07-1.68)	0.01

Values are number (%), and OR (95%CI).

BMI=body mass index; CI=Confidence interval; HF=heart failure; LVEF=left ventricular ejection fraction; OR=odds ratio; TR=tricuspid regurgitation.

Supplemental Table S2. Detailed Data on Baseline Characteristics According to the Status of Anemia

Variables	No anemia<br>Hb ≥13.0 g/dl for men, and<br>≥12.0 g/dl for women<br>N=1286	Mild anemia<br>Hb 11.0–12.9 g/dl for men,<br>and 11.0–11.9 g/dl for women<br>N=835	Moderate/Severe anemia<br>Hb ≤10.9 g/dl<br>N=1282	P value
**Clinical characteristics**				
Age, y* †	74.5 ± 9.9	78.0 ± 8.7	81.4 ± 8.7	<0.001
Age ≥80	393 (31)	372 (45)	792 (62)	<0.001
Men*	524 (41)	399 (48)	371 (29)	<0.001
BMI, kg/m^2	22.7 ± 3.7	22.0 ± 3.7	20.6 ± 3.6	<0.001
BMI <22 kg/m^2*	634 (49)	491 (59)	941 (73)	<0.001

Hypertension*	879 (68)	598 (72)	923 (72)	0.09
Current smoking*	87 (7)	51 (6)	37 (3)	<0.001
History of smoking	323 (25)	222 (27)	210 (16)	<0.001
Dyslipidemia	514 (40)	308 (37)	372 (29)	<0.001
On statin therapy	353 (27)	238 (29)	288 (22)	0.002
Diabetes mellitus	296 (23)	209 (25)	323 (25)	0.4
On insulin therapy*	39 (3)	59 (7)	78 (6)	<0.001
Coronary artery disease*	341 (27)	304 (36)	398 (31)	<0.001
Prior PCI	126 (10)	132 (16)	189 (15)	<0.001
Prior CABG	38 (3)	58 (7)	72 (6)	<0.001
Prior myocardial infarction*	73 (6)	78 (9)	132 (10)	<0.001

Prior open heart surgery	73 (6)	82 (10)	111 (9)	<0.001
Prior HF	158 (12)	119 (14)	314 (24)	<0.001
Prior symptomatic stroke*	166 (13)	128 (15)	174 (14)	0.30
Atrial fibrillation or flutter* †	270 (21)	172 (21)	311 (24)	0.06
Aortic/peripheral vascular disease*	156 (12)	152 (18)	227 (18)	<0.001
Serum creatinine, mg/dl*	0.8 (0.6–1.0)	0.9 (0.7–1.2)	1.1 (0.8–2.3)	<0.001
Creatinine >2 mg/dl	40 (3)	124 (15)	360 (28)	<0.001
eGFR, ml/min/1.73m^2	64 ± 22	51 ± 26	39 ± 27	0.001
Hemodialysis*	36 (3)	105 (13)	230 (18)	<0.001
Hemoglobin, g/dl	13.4 (12.7–14.2)	11.6 (11.3–11.9)	9.7 (8.7–10.4)	<0.001
BNP, pg/ml	143 (57–432)	216 (97–615)	554 (202–1357)	<0.001

CRP, mg/dl	0.13 (0.06–0.36)	0.2 (0.08–0.64)	0.43 (0.1–2.2)	<0.001
Liver cirrhosis (Child-Pugh B or C)*	8 (1)	8 (1)	21 (2)	0.04
Malignancy	145 (11)	114 (14)	216 (17)	<0.001
Malignancy currently under treatment* †	33 (3)	39 (5)	71 (6)	<0.001
Chronic lung disease	159 (12)	88 (11)	119 (9)	0.04
Chronic lung disease (moderate or severe)*	41 (3)	21 (3)	43 (3)	0.5
Logistic EuroSCORE, %	7.0 (4.2–12.0)	9.4 (6.2–16.0)	14.2 (9.0–23.0)	<0.001
EuroSCORE II, %	1.9 (1.2–3.5)	2.8 (1.7–4.5)	4.1 (2.7–6.8)	<0.001
STS score (PROM), %	2.5 (1.6–4.0)	3.8 (2.4–5.9)	6.1 (3.7–1.0)	<0.001
**Echocardiographic variables**				
Vmax, m/s	4.2±0.9	4.2±0.9	4.1±0.9	0.01

Vmax >5 m/s	261 (20)	171 (20)	231 (18)	0.2
Vmax >4 m/s* †	790 (61)	491 (59)	713 (56)	0.01
Mean aortic PG, mmHg	42±20	42±21	41±20	0.09
AVA (equation of continuity), cm^2	0.73±0.18	0.72±0.18	0.69±0.19	<0.001
Low gradient AS (Vmax ≤4m/s and mean aortic PG ≤40mmHg, but AVA <1.0 cm^2)	537 (42)	356 (43)	597 (47)	0.04
LV end-diastolic diameters, mm	46±7	46±7	46±7	0.5
LV end-systolic diameters, mm	30±8	30±8	31±8	<0.001
LVEF, %*	64±13	63±13	61±14	<0.001
<40%	90 (7)	57 (7)	133 (10)	0.002
<50%	178 (14)	117 (14)	263 (21)	<0.001

IVST in diastole, mm	11.5±2.4	11.5±2.3	11.2±2.2	0.002
PWT in diastole, mm	11.1±2.0	11.1±2.2	10.9±2.0	0.04
Any combined valvular disease (moderate or severe)*†	475 (37)	324 (39)	623 (49)	<0.001
Moderate or severe AR	280 (22)	174 (21)	263 (21)	0.7
Moderate or severe MS	51 (4)	24 (3)	46 (4)	0.4
Moderate or severe MR	191 (15)	152 (18)	362 (28)	<0.001
Moderate or severe TR	159 (12)	124 (15)	289 (23)	<0.001
TR pressure gradient ≥40 mm Hg*	158 (12)	117 (14)	292 (23)	<0.001
**Symptomatic status at index UCG**				
Chest pain	176 (14)	142 (17)	154 (12)	0.02

Syncope	66 (5)	53 (6)	74 (6)	0.5
HF	494 (38)	302 (36)	727 (57)	<0.001
NYHA III or IV	172 (35)	127 (42)	414 (57)	<0.001
Asymptomatic	637 (50)	407 (49)	455 (35)	<0.001
Admission for HF at index echocardiography* †	194 (15)	123 (15)	454 (35)	<0.001
**Therapeutic strategy***				
Initial AVR	549 (43)	316 (38)	313 (24)	<0.001
Conservative	737 (57)	519 (62)	969 (76)	<0.001

Values are mean ± SD, median (interquartile range), or number (%).

The values of CRP and BNP were obtained in 2914 (76%) and 1801 (47%) patients, respectively. The NYHA class was obtained in 1500 (44.1%) patients.

* † Potential risk-adjusting variables selected for the Cox proportional hazards models in the entire population (*) and in the stratified analyses across the initial AVR/conservative strata (†).

AR=aortic regurgitation; AS=aortic stenosis; AVA=aortic valve area; AVR=aortic valve replacement; BMI=body mass index; BNP= brain-derived natriuretic peptide; CABG=coronary artery bypass grafting; CRP=C-reactive protein; eGFR=estimate glomerular filtration rate; Hb=hemoglobin; HF=heart failure; IVST=interventricular septum thickness, LV=left ventricular; LVEF=left ventricular ejection fraction; MR=mitral regurgitation; MS=mitral stenosis; NYHA=New York Heart Association; PCI=percutaneous coronary intervention; PG=pressure gradient; PROM=predicted risk of mortality; PWT=posterior wall thickness; STS=Society of Thoracic Surgeons; TR=tricuspid regurgitation; UCG=ultrasound echocardiography; and Vmax=peak aortic jet velocity.

Supplemental Table S3. List of adjusting variable in the Cox proportional hazard models

**Adjusting variables in the Cox proportional hazard models**	**Adjusting variables in the parsimonious Cox proportional hazard models**
Age	Age
Men	Vmax >4m/s
BMI <22 kg/m2	Any combined valvular disease (moderate or severe)
Therapeutic strategy	Atrial fibrillation or flutter
LVEF	Malignancy currently under treatment
TR pressure gradient ≥40 mmHg	Admission for HF at index echocardiography
Vmax >4m/s	
Any combined valvular disease (moderate or severe)	

Prior myocardial infarction

Prior symptomatic stroke

Atrial fibrillation or flutter

Chronic lung disease (moderate or severe)

Malignancy currently under treatment

Aortic/peripheral vascular disease

Liver cirrhosis (Child-Pugh B or C)

Hemodialysis

Admission for HF at index echocardiography

Hypertension

Current smoking

On insulin therapy	
Coronary artery disease	
Serum creatinine	

BMI=body mass index; HF=heart failure; LVEF=left ventricular ejection fraction; TR=tricuspid regurgitation; Vmax=peak aortic jet velocity.

Supplemental Figure S1

A.

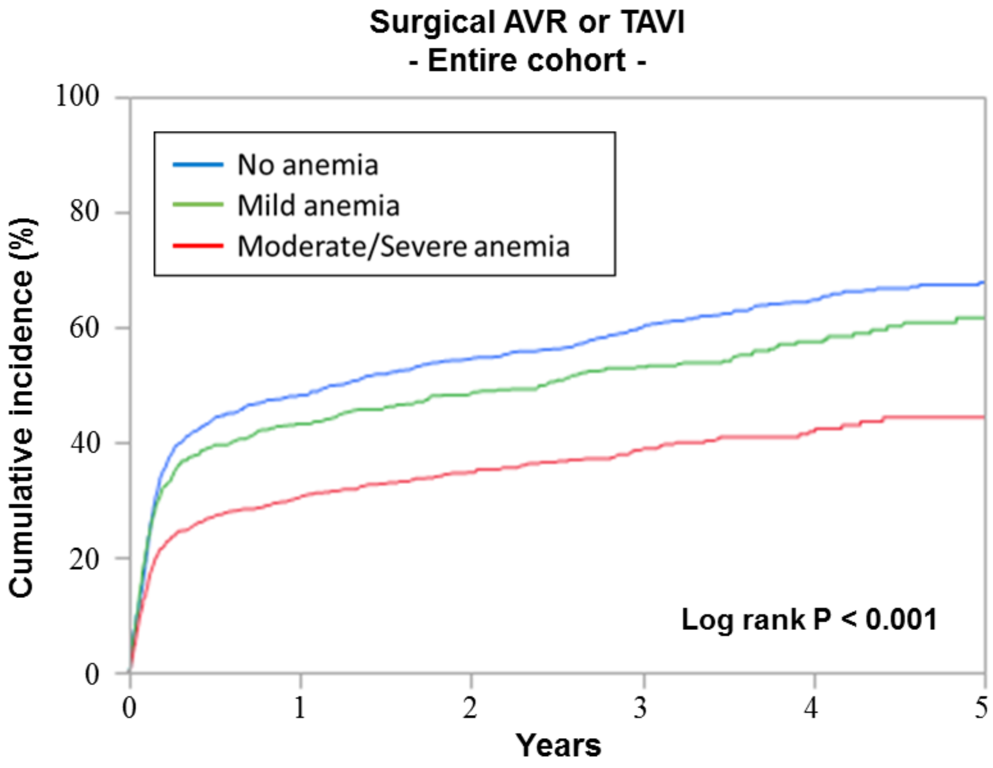

	0	1 year	2 year	3 year	4 year	5 year
No anemia						
N of patients at risk	1286	585	458	309	180	81
N of patients with event		597	667	715	745	757
Cumulative incidence		48%	55%	60%	65%	68%
Mild anemia						
N of patients at risk	835	385	297	198	96	42
N of patients with event		342	375	398	411	418
Cumulative incidence		43%	49%	53%	57%	62%
Moderate/Severe anemia						
N of patients at risk	1282	573	390	236	114	50
N of patients with event		349	380	400	409	413
Cumulative incidence		30%	35%	39%	42%	44%

B.

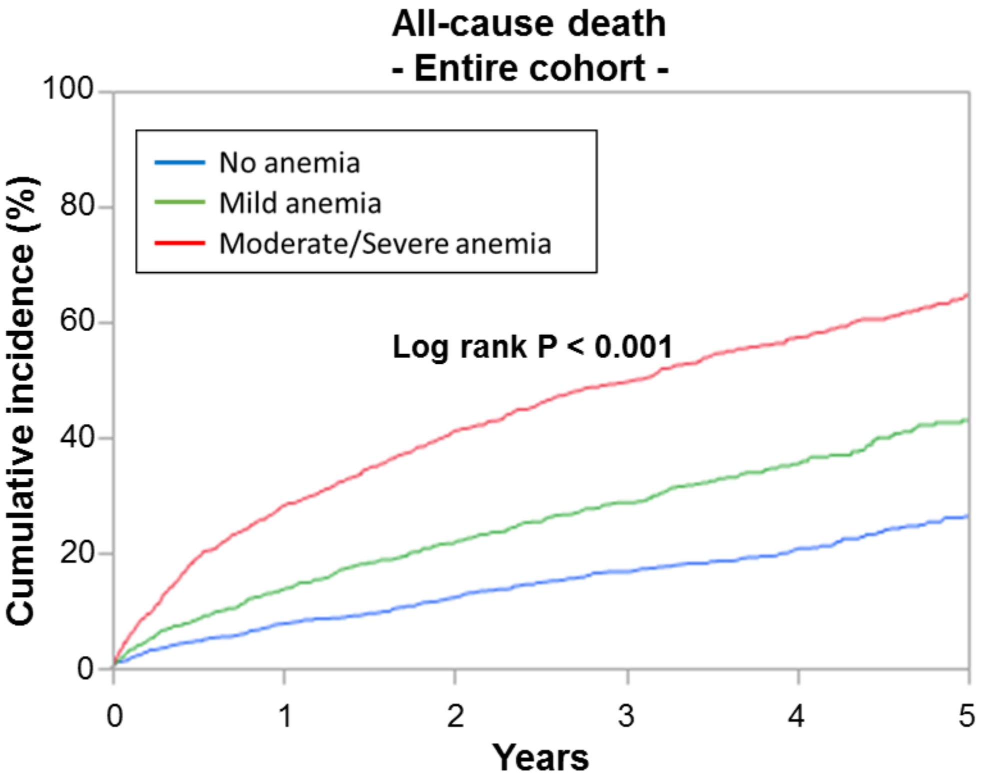

	0	1 year	2 year	3 year	4 year	5 year
**No anemia**						
N of patients at risk	1286	1144	1061	808	517	279
N of patients with event		98	153	203	234	263
Cumulative incidence		8%	12%	17%	21%	26%
**Mild anemia**						
N of patients at risk	835	685	598	439	259	134
N of patients with event		111	175	224	258	282
Cumulative incidence		14%	22%	29%	35%	43%
**Moderate/Severe anemia**						
N of patients at risk	1282	857	672	459	239	114
N of patients with event		345	499	588	646	677
Cumulative incidence		28%	41%	50%	57%	65%

C.

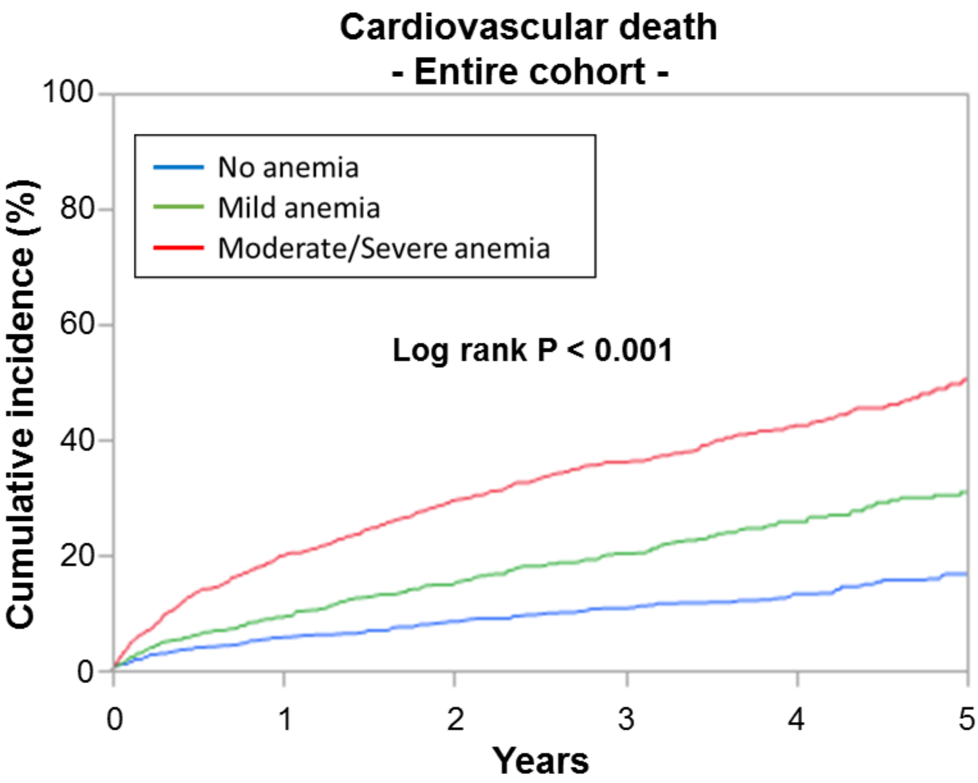

	0	1 year	2 year	3 year	4 year	5 year
**No anemia**						
N of patients at risk	1286	1144	1061	808	517	279
N of patients with event		72	105	129	147	163
Cumulative incidence		6%	9%	11%	13%	17%
**Mild anemia**						
N of patients at risk	835	685	598	439	259	134
N of patients with event		73	117	150	175	189
Cumulative incidence		9%	15%	20%	26%	31%
**Moderate/Severe anemia**						
N of patients at risk	1282	857	672	459	239	114
N of patients with event		233	332	388	424	448
Cumulative incidence		20%	30%	36%	42%	50%

D.

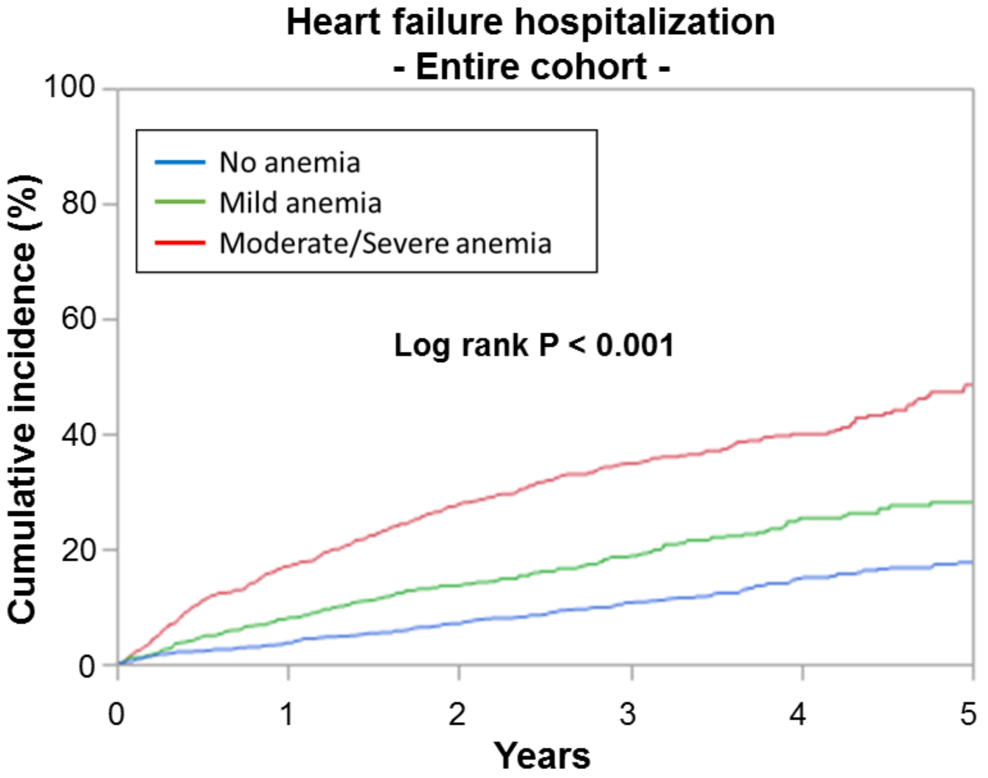

	0	1 year	2 year	3 year	4 year	5 year
No anemia						
N of patients at risk	1286	1114	1011	751	466	256
N of patients with event		45	73	35	146	158
Cumulative incidence		4%	7%	11%	15%	18%
Mild anemia						
N of patients at risk	835	648	546	385	225	118
N of patients with event		61	99	127	152	158
Cumulative incidence		8%	14%	19%	25%	28%
Moderate/Severe anemia						
N of patients at risk	1282	770	568	367	181	78
N of patients with event		179	273	322	344	362
Cumulative incidence		17%	28%	35%	40%	48%

E.

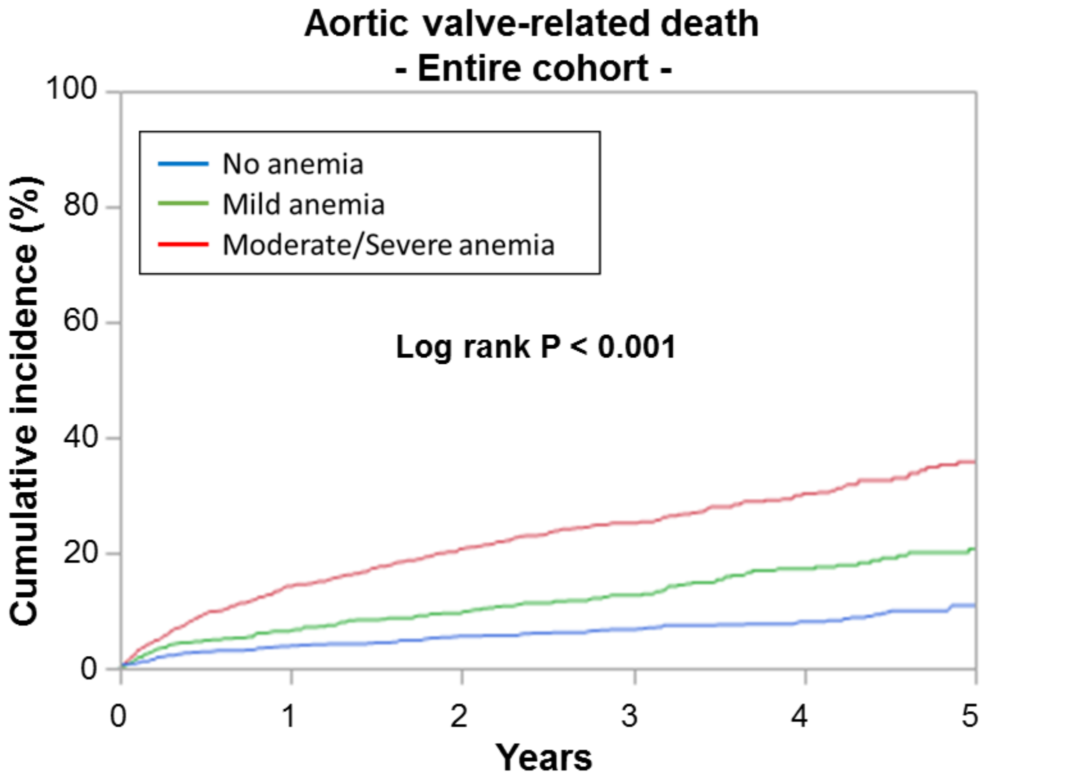

	0	1 year	2 year	3 year	4 year	5 year
No anemia						
N of patients at risk	1286	1144	1062	808	517	279
N of patients with event		48	68	79	89	101
Cumulative incidence		4%	6%	7%	8%	11%
Mild anemia						
N of patients at risk	835	685	598	439	260	134
N of patients with event		51	74	92	111	119
Cumulative incidence		6%	10%	13%	17%	21%
Moderate/Severe anemia						
N of patients at risk	1282	857	672	459	239	114
N of patients with event		161	222	255	279	293
Cumulative incidence		14%	21%	25%	30%	36%

F.

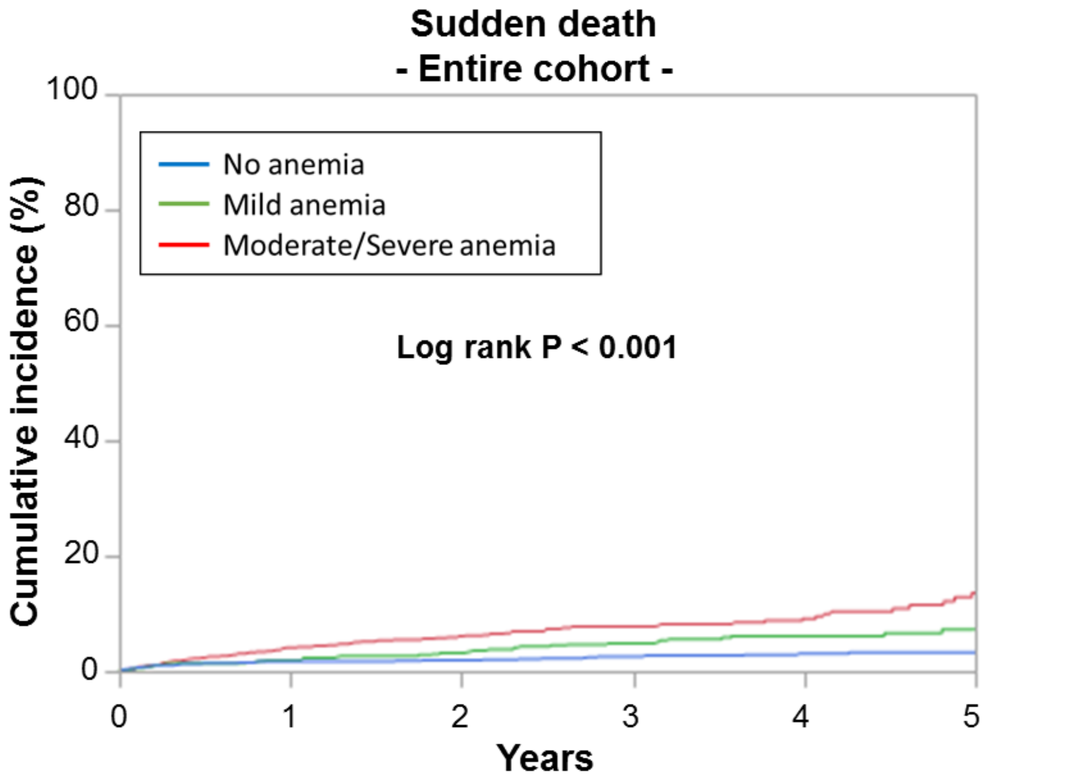

	0	1 year	2 year	3 year	4 year	5 year
No anemia						
N of patients at risk	1286	1144	1062	808	517	279
N of patients with event		21	24	30	33	34
Cumulative incidence		2%	2%	3%	3%	3%
Mild anemia						
N of patients at risk	835	685	598	439	260	134
N of patients with event		15	23	33	38	40
Cumulative incidence		2%	3%	5%	6%	7%
Moderate/Severe anemia						
N of patients at risk	1282	857	672	459	239	114
N of patients with event		43	60	70	75	83
Cumulative incidence		4%	6%	8%	9%	14%

G.

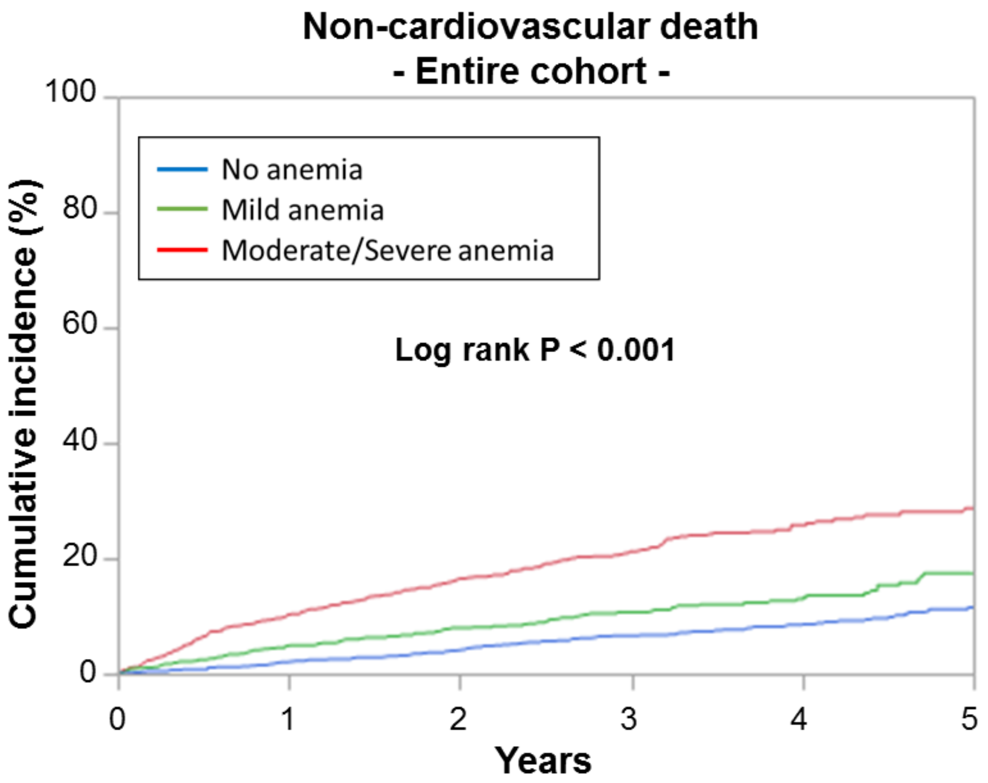

	0	1 year	2 year	3 year	4 year	5 year
No anemia						
N of patients at risk	1286	1144	1062	808	517	279
N of patients with event		26	48	74	87	100
Cumulative incidence		2%	4%	7%	8%	12%
Mild anemia						
N of patients at risk	835	685	598	439	259	134
N of patients with event		38	58	74	83	93
Cumulative incidence		5%	8%	11%	13%	17%
Moderate/Severe anemia						
N of patients at risk	1282	857	672	459	239	114
N of patients with event		112	167	200	222	229
Cumulative incidence		10%	17%	21%	26%	29%

Supplemental Figure S2

Variables	Subpopulation	Severity of anemia	Cumulative 5-year incidence/Number at risk (Cumulative 5-year event rate %)	Unadjusted HR (95% CI)	P value	Adjusted HR (95%CI)	P value	Adjusted HR (95% CI)	P for interaction
**Age**	Age <80	No	109/893 (16%)	1 (reference)		1 (reference)			0.09
		Mild	95/463 (26%)	1.75 (1.35-2.27)	<0.001	1.06 (0.8-1.41)	0.7		
		Moderate/Severe	150/490 (42%)	3.29 (2.61-4.17)	<0.001	1.65 (1.25-2.18)	<0.001		
	Age ≥80	No	100/393 (38%)	1 (reference)		1 (reference)			
		Mild	119/372 (48%)	1.34 (1.04-1.74)	0.02	1.46 (1.12-1.92)	0.006		
		Moderate/Severe	332/792 (68%)	2.23 (1.8-2.78)	<0.001	1.52 (1.19-1.93)	<0.001		
**AS-related symptoms**	Asymptomatic	No	78/638 (17%)	1 (reference)		1 (reference)			0.5
		Mild	98/408 (34%)	2.11 (1.6-2.79)	<0.001	1.57 (1.16-2.12)	0.004		
		Moderate/Severe	117/455 (41%)	2.92 (2.23-3.84)	<0.001	1.88 (1.38-2.57)	<0.001		
	Symptomatic	No	131/648 (26%)	1 (reference)		1 (reference)			
		Mild	116/427 (34%)	1.46 (1.15-1.86)	0.002	1.12 (0.87-1.45)	0.4		
		Moderate/Severe	365/827 (64%)	3.12 (2.58-3.8)	<0.001	1.43 (1.14-1.79)	0.002		
**Severity of AS**	Vmax <5m/s	No	177/1025 (24%)	1 (reference)		1 (reference)			1
		Mild	178/664 (37%)	1.74 (1.42-2.12)	0.005	1.34 (1.09-1.65)	0.006		
		Moderate/Severe	398/1051 (58%)	3.24 (2.73-3.84)	<0.001	1.63 (1.34-1.99)	<0.001		
	Vmax ≥5m/s	No	32/261 (14%)	1 (reference)		1 (reference)			
		Mild	36/171 (26%)	1.7 (1.08-2.69)	0.02	1.17 (0.7-1.97)	0.6		
		Moderate/Severe	84/231 (47%)	3.72 (2.54-5.55)	<0.001	1.33 (0.83-2.13)	0.2		
**LV systolic function**	EF ≥50%	No	164/1108 (20%)	1 (reference)		1 (reference)			0.3
		Mild	167/718 (32%)	1.65 (1.35-2.03)	<0.001	1.24 (1.002-1.53)	0.048		
		Moderate/Severe	354/1019 (52%)	3.31 (2.78-3.95)	<0.001	1.62 (1.32-1.98)	<0.001		
	EF <50%	No	45/178 (32%)	1 (reference)		1 (reference)			
		Mild	47/117 (48%)	1.99 (1.33-2.99)	<0.001	1.48 (0.93-2.34)	0.09		
		Moderate/Severe	128/263 (71%)	2.86 (2.05-4.06)	<0.001	1.52 (1-2.3)	0.0501		
**Renal function**	CRE ≤2mg/dl	No	192/1246 (21%)	1 (reference)		1 (reference)			<0.001
		Mild	175/711 (33%)	1.68 (1.38-2.04)	<0.001	1.41 (1.15-1.72)	<0.001		
		Moderate/Severe	337/922 (54%)	3.31 (2.80-3.93)	<0.001	1.73 (1.42-2.1)	<.0001		
	CRE >2mg/dl or hemodialysis	No	17/40 (52%)	1 (reference)		1 (reference)			
		Mild	39/124 (44%)	0.66 (0.38-1.19)	0.2	0.4 (0.22-0.77)	0.005		
		Moderate/Severe	145/360 (63%)	0.96 (0.60-1.65)	0.9	0.5 (0.3-0.95)	0.03		

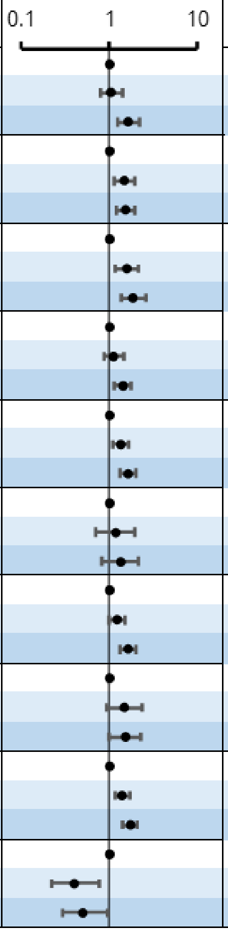

Supplemental Figure S3

A.

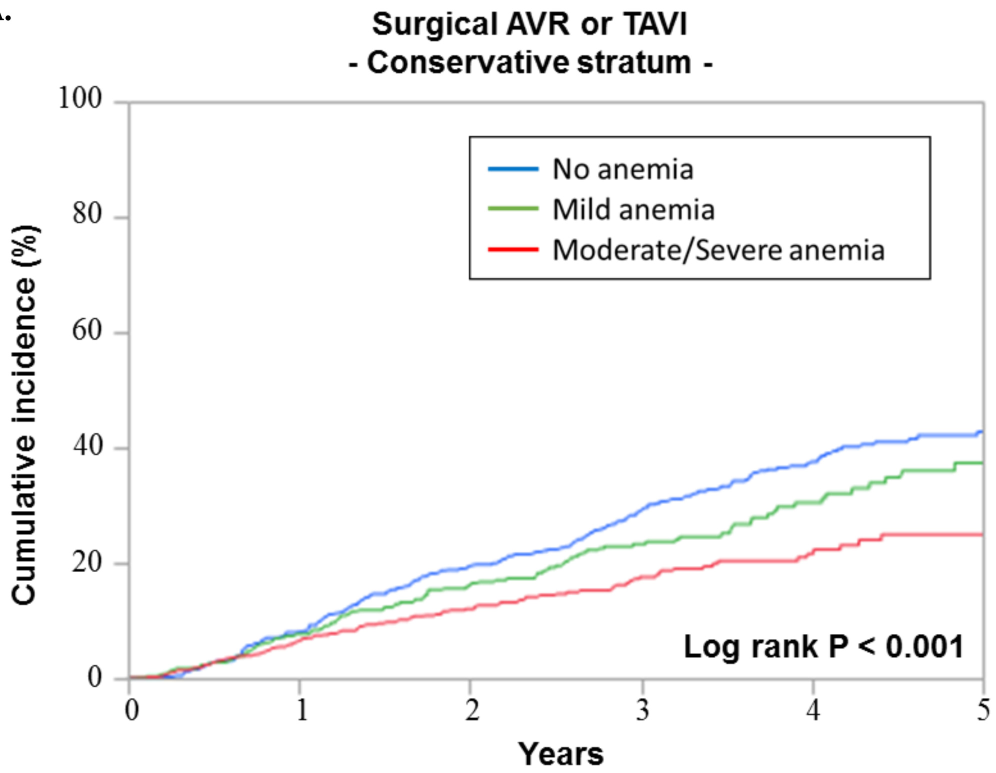

	0	1 year	2 year	3 year	4 year	5 year
**No anemia**						
N of patients at risk	737	584	457	308	180	81
N of patients with event		54	124	172	202	214
Cumulative incidence		8%	20%	29%	38%	43%
**Mild anemia**						
N of patients at risk	519	382	295	197	95	42
N of patients with event		34	67	89	102	109
Cumulative incidence		8%	16%	23%	30%	37%
**Moderate/Severe anemia**						
N of patients at risk	969	569	388	234	114	50
N of patients with event		46	76	96	105	109
Cumulative incidence		6%	12%	18%	22%	25%

B.

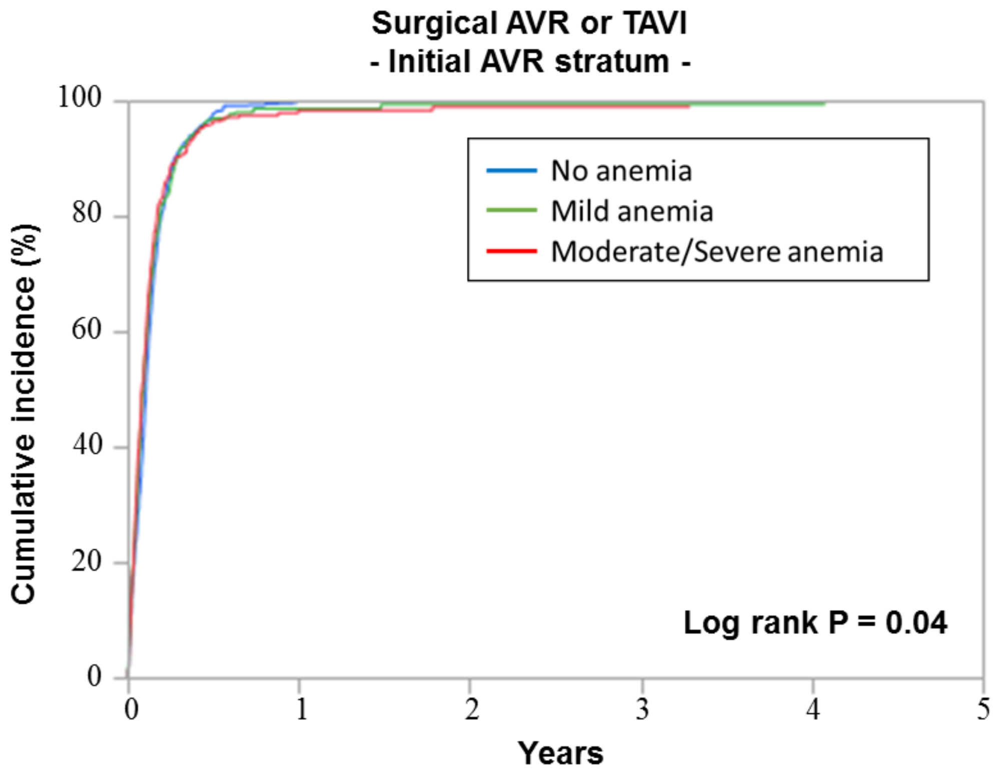

	0	1 year
No anemia		
N of patients at risk	549	1
N of patients with event		543
Cumulative incidence		100%
Mild anemia		
N of patients at risk	316	2
N of patients with event		308
Cumulative incidence		99%
Moderate/Severe anemia		
N of patients at risk	313	4
N of patients with event		303
Cumulative incidence		98%

Supplemental Figure Legends

Supplemental Figure S1

Cumulative incidences of surgical AVR or TAVI and the secondary outcome measures in the no, mild, and moderate/severe anemia groups in the entire cohort.

AVR=aortic valve replacement; TAVI=transcatheter aortic valve implantation

Supplemental Figure S2

Subgroup analyses for the crude risks of mild and moderate/severe anemia relative to no anemia for the primary outcome measure.

AS=aortic stenosis; EF=ejection fraction; eGFR=estimated glomerular filtration rate; HR=hazard ratio; LV=left ventricular

Supplemental Figure S3

Kaplan–Meier curves for the cumulative incidences of surgical AVR or TAVI in the conservative stratum (A) and in the initial AVR stratum (B).

AVR=aortic valve replacement; TAVI=transcatheter aortic valve implantation
